# Effect of topical rebamipide on conjunctival goblet cell recovery after vitrectomy

**DOI:** 10.1038/srep19516

**Published:** 2016-01-14

**Authors:** Kumiko Kato, Yuko Takashima, Koichi Matsunaga, Masahiko Sugimoto, Hisashi Matsubara, Koji Hirano, Mineo Kondo

**Affiliations:** 1 Department of Ophthalmology, Mie University School of Medicine, Tsu, Japan; 2 Matsunaga Eye Clinic, Tsu, Japan; 3 Department of Ophthalmology, Ban Buntane Hotokukai Hospital, School of Medicine, Fujita Health University, Nagoya, Japan

## Abstract

*In vitro* and *in vivo* experiments have shown that topical rebamipide will increase the number of goblet cells in the bulbar conjunctiva. The purpose of this study was to determine whether topical rebamipide will enhance the recovery of conjunctival goblet cells that were damaged during vitrectomy. Forty patients who underwent vitrectomy surgery were studied. The 40 patients consisted of 20 with diabetes mellitus (DM) and 20 patients without DM. They were randomized in a 1:1 ratio into groups that were treated or not treated with topical 2% rebamipide after the surgery. Impression cytology was performed at the end of surgery and at 14 days after the surgery. The mean goblet cell density of each specimen was determined by averaging the total number of goblet cells obtained from three consecutive high magnification microscopic images. In patients without DM, the mean goblet cell density at 14 days after the vitrectomy was significantly higher in eyes with topical rebemipide than in eyes without rebemipide (*P* < 0.01). In patients with DM, a similar tendency was observed but the difference was not significant (*P* = 0.09). These results suggest that topical rebamipide can be helpful in patients with globlet cell damage that occur during and after vitrectomy.

Recent advances in ophthalmic surgery have markedly improved patients’ postoperative vision. However, these benefits can be reduced if the ocular surface is damaged before, during, and after the surgery, and patients often complain of dry eyes after refractive or cataract surgeries^1^,^2^,^3^,^4^,^5^,^6^. The damages of the ocular surface can be caused by various factors including preoperative disinfection solutions, dessication of the ocular surface during the surgery, intra- or postoperative inflammation, injury of ocular surface nerves by the surgery, and epithelial toxicity by topical postoperative medications.

Relatively severe ocular surface damage can occur during vitrectomy probably because of the longer operation time and the more invasive surgical procedures^7^,^8^. Heimann *et al.*^9^ examined biopsy specimens of the bulbar conjunctiva about 11 months after vitrectomy and reported an increase in the epithelial stratification and a significant decrease in the number of PAS- and MUC5AC-positive goblet cells. They suggested that vitrectomy can lead to morphological alterations of the conjunctiva and distribution of ocular mucins which can then cause dry eye symptoms^9^. Such changes in the ocular surface can be more severe when patients have diabetes mellitus (DM)^10^,^11^ because the healing of epithelial wounds is delayed in eyes with DM^12^,^13^,^14^,^15^.

To minimize the ocular surface damage and dry eye symptoms after the surgery, various pre- or postoperative treatments have been proposed. These treatments include the postoperative use of unpreserved artificial tears, autologous serum eye drops^16^, gels or ointments, hot compresses and/or eyelid scrubs, nutritional supplements, steroid, or other anti-inflammatory drugs^17^. However, these treatments are still not necessarily satisfactory, and new methods are needed.

Rebamipide is a quinolinone derivative with mucin secretagogue activity and is widely used as an antigastric ulcer drug^18^. It is known that the *in vivo* application of topical rebamipide increases both the number of goblet cells and the level of mucin-like substances in the bulbar conjunctiva of rabbits^19^,^20^, increases the degree of proliferation of cultured rat conjunctival goblet cells^21^, and induces mucin secretion in cultured conjunctival goblet cells of rat^22^. Recent clinical trials have shown that a topical rebamipide ophthalmic suspension is effective in improving both the signs and symptoms of dry eye patients^23^,^24^,^25^. These observations suggested that topical rebamipide might enhance the recovery of conjunctival goblet cells that are damaged during and after vitrectomy.

Thus, the purpose of this study was to determine whether topical rebamipide will increase the number of conjunctival goblet cells after the vitrectomy. To accomplish this, we used impression cytology immediately and 14 days after vitrectomy in patients with and without DM who were either treated or not treated with topical rebamipide.

## Methods

This was a prospective, randomized comparative study conducted at the Mie University Hospital between December 2013 and April 2014. The procedures used in this study were approved by the Medical Ethics Committee of Mie University Hospital (No. 2617). The procedures conformed to the tenets of the Declaration of Helsinki of the World Medical Association. All patients signed a written informed consent after they were provided with sufficient information on the procedures to be used. The study was registered International Clinical Trial Registry Platform (UMIN000015888; registered December 15, 2014).

Forty eyes of 40 patients consisting of 20 with DM and 20 without DM who required vitrectomy were studied. The exclusion criteria included; prior keratoconjunctival diseases, other intraocular surgeries within the past six months, and use of any topical medications before the surgery.

### Surgery and randomization

Vitrectomy was performed by three experienced vitreoretinal surgeons (MK, HM, and MS) in the Mie University School of Medicine Hospital. After local anesthesia by sub-Tenon capsule injection of lidocaine hydrochloride, standard 4 port pars plana vitrectomy was performed using a 23-gauge or 25-gauge microincision instruments with chandelier illumination. The Constellation® Vision System (Alcon, Irvine, CA, USA) was used for all of the surgeries. There was no vitrectomy port or incision on the bulbar conjunctiva lower than 90 degrees where the impression cytology was to be performed. For additional anesthesia during the surgery, 2% lidocaine and 0.2% ropivacaine were applied as eye drops or sub-Tenon injections.

After the surgery, all eyes received three drops each of 0.5% moxifloxacin (Alcon Inc., Tokyo, Japan), 0.1% bethamethason (Shionogi & Co., Ltd. Osaka, Japan), and 0.1% nepafenac (Alcon Inc., Tokyo, Japan). These treatments were begun one day after the vitrectomy and performed three times daily until 14 days after the surgery.

**Figure 1:**
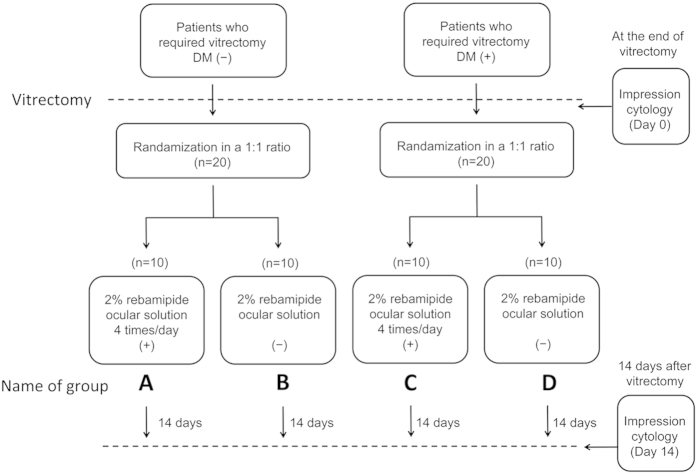
Study design. Forty eyes of 40 patients consisting of 20 with diabetes mellitus (DM) and 20 without DM who underwent vitrectomy were studied. After the vitrectomy, the 20 eyes with DM and 20 eyes without DM were further randomized sequentially in a 1:1 ratio to treatment with or without 2% rebamipide ocular solution four times/day. Impression cytology was performed at the end of surgery and at 14 days after the vitrectomy. DM, diabetes mellitus.

After the vitrectomy surgery, 20 eyes without DM and 20 eyes with DM were further randomized sequentially in a 1:1 ratio to postoperative treatment with 2% rebamipide ocular solution four times daily or without rebamipide (Fig. 1). Group A consisted of eyes of 10 patients without DM who received rebamipide, Group B consisted of 10 eyes of patients without DM who did not receive rebamipide, Group C consisted of 10 eyes of patients with DM who received topical rebamipide, and Group D consisted of 10 eyes of patients with DM who did not receive rebamipide. In Groups A and C, the 2% topical rebamipide ocular solution was started one day after the vitrectomy and performed four times daily until 14 days after the surgery.

### Impression cytology

Impression cytology^26^ was performed under topical anesthesia at the completion of vitrectomy and again on day 14 after the surgery. For the impression cytology, nitrocellulose membrane strips with 0.22 μm pore size (Merck Millipore Ltd, Darmstade, Germany) were placed on the inferior bulbar conjunctiva. The strips remained in contact with the conjunctiva for approximately 10 seconds and then peeled off with forceps. All strips were placed on microscopic glass slides and stained with periodic acid-Schiff (PAS). The strips were mounted with Entellan (Merck, Darmstadt, Germany) and were examined and photographed under a light microscope at a magnification of 200x. The mean density of goblet cells of each specimen was determined by averaging the total number of goblet cells in three consecutive fields of magnification 200×.

### Statistical analyses

Unpaired *t* tests, Chi-squared tests, and Fisher’s exact tests were used to determine if the differences in the background or surgical factors between Groups A and B or between Groups C and D were significant (Tables 1 and 2). Paired *t* tests were also performed to determine if the density of goblet cells immediately after the vitrectomy was significantly different from the density 14 days after the vitrectomy within the same group. Unpaired *t* tests were used to examine if the differences in the density of the goblet cells between two groups were significant. Results were considered statistically significant when *P* < 0.05.

## Results

The demographic data of the four groups are presented in Table 1. There was no significant difference in the age, operation time, or frequency of combined PEA + IOL between Groups A and B or between Groups C and D. There was also no significant difference in the level of HbA1c or duration of the DM between Groups C and D. There were significant differences only in the sex distribution between Groups A and B and between Groups C and D. There were no significant differences in the type of retinal diseases that necessitated the vitrectomy between Groups A and B and Groups C and D (Table 2).

**Figure 2:**
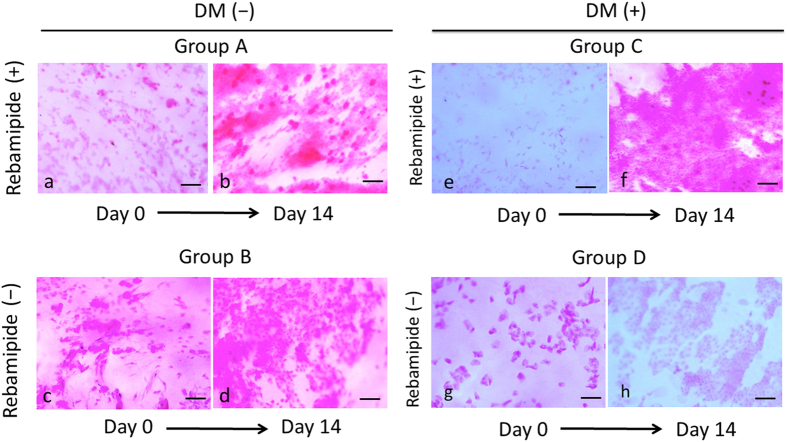
Impression cytology photomicrographs (PAS staining) of specimens obtained from the eyes of representative patients in the four groups (A–D). Impression cytology images just after the vitrectomy (Day 0) and 14 days after the vitrectomy (Day 14) in the same subjects are shown. Group A are the eyes that had topical rebamipide in patients without DM. Group B are the eyes that did not receive rebamipide in patients without DM. Group C are the eyes that received rebamipide in patients with DM. Group D are the eyes that did not receive rebamipide in patients with DM. Scale bars, 50 μm.

Images of the impression cytology obtained from the eyes of representative patients from the four groups are shown in Fig. 2. These impression cytology images were obtained from specimens collected immediately after the vitrectomy (Day 0) and 14 days after the vitrectomy (Day 14) from the same subjects. The mean (±SD) density of goblet cells at the end of vitrectomy in eyes without DM (Groups A and B) was 40.8 ± 48.6 number/mm^2^ (n = 20), which was significantly higher than that in eyes with DM at 15.4 ± 24.5 count/mm^2^ (*P* = 0.04; n = 20). The mean (±SD) density of goblet cells in normal patients of our institute is 314.2 ± 176.8 number/mm^2^ (age, 71.2 ± 6.2 years; n = 10).

**Figure 3:**
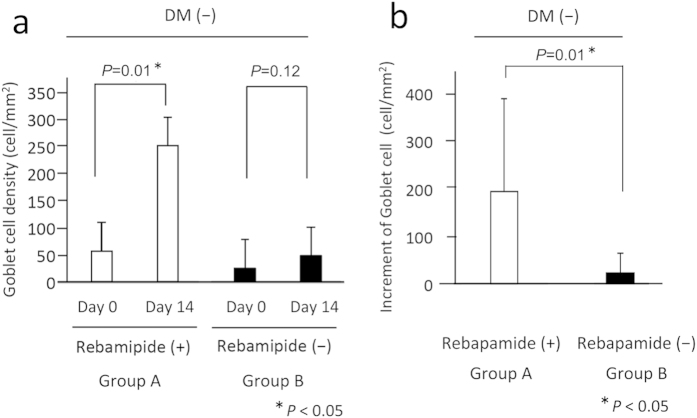
Graph of means and standard deviations of cell numbers immediately after vitrectomy and at 14 days after vitrectomy. (**a**) Goblet cell number at the end of vitrectomy (Day 0) and at 14 days after the vitrectomy (Day 14) in Groups A and B. (**b**) The increase of goblet cell density during the 14 days after the vitrectomy in Groups A and B. The increase of goblet cell density during the 14 days after the vitrectomy was significantly higher in Group A than in Group B (*P* < 0.01, unpaired *t* tests).

The mean (±SD) density of goblet cells just after the vitrectomy in Group A was 56.5 ± 57.3 number/mm^2^, and it increased significantly to 250.7 ± 186.9 number/mm^2^ at 14 days after the vitrectomy (*P* = 0.01, left panel of Fig. 3a). In Group B, the mean density of goblet cells just after the vitrectomy was 25.1 ± 33.9 number/mm^2^, and it increased to 47.7 ± 49.3 number/mm^2^ at 14 days after the vitrectomy. This difference was not statistically significant (*P* = 0.12, right panel of Fig. 3a). Because the mean number of goblet cells just after the vitrectomy (Day 0) was not significantly different between Groups A and B, we compared the increase in the number of goblets cell at 14 days after the vitrectomy between the two groups. We found that the increase in the mean number of goblet cells was significantly higher in Group A (194.2 ± 194.4 number/mm^2^) than in Group B (22.6 ± 41.7 number/mm^2^, *P* = 0.01, Fig. 2b).

**Figure 4:**
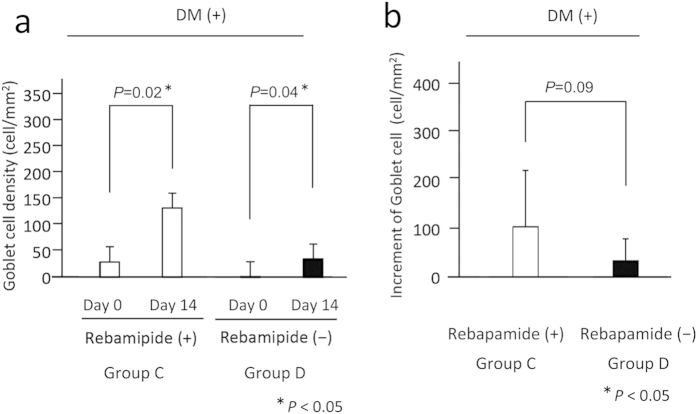
Graph of means and standard deviations of cell numbers immediately after vitrectomy and at 14 days after vitrectomy. (**a**) The goblet cell number at the end of vitrectomy (Day 0) and at 14 days after the vitrectomy (Day 14) in Groups C and D. (**b**) Increase in goblet cell density at 14 days after the vitrectomy in Groups C and D. The increase in the goblet cell density during 14 days after the vitrectomy was higher in Group C than in Group D but the difference was not statistically significant (*P* = 0.09, unpaired *t* tests).

Next, we compared the density of goblet cells in the patients with DM (Groups C and D). In Group C, the mean density of goblet cells just after the vitrectomy was 29.1 ± 29.0 number/mm^2^, and it increased significantly to 132.4 ± 118.5 number/mm^2^ at 14 days after the vitrectomy (*P* = 0.02, left panel of Fig. 4a). In the Group D, the mean number of goblet cells just after the vitrectomy was 1.7 ± 2.7 cells/mm^2^ (n = 10), and it also increased significantly to 35.0 ± 46.5 cells/mm^2^ 14 days after the vitrectomy (*P* = 0.04, right panel of Fig. 4a). Again because the density of goblet cells just after the vitrectomy was not the same between Groups C and D, we compared the increase in the number of goblet cells during the 14 days after the vitrectomy between the two groups. The mean increase in the goblet cell density during the 14 days in Group C was 103.3 ± 116.7 cells/mm^2^ which was higher than that in Group D at 33.3 ± 45.6 cells/mm^2^ but the difference was not statistically significant (*P* = 0.09).

We also assessed the corneal status by fluorescein corneal staining score before and after the surgery, and compared the scores between Group A and B, and between Group C and D. However, there was no significant difference.

## Discussion

Our results demonstrated that topical rebamipide promotes the recovery of conjunctival goblet cells after vitrectomy. In eyes of non-DM patients, the increase in the density of goblet cells measured by impression cytology was significantly higher in eyes treated with topical rebamipide than in eyes without rebamipide (Fig. 3). In eyes of DM patients, a similar increase was found but the difference was not statistically significant (Fig. 4).

The mechanisms of how topical rebamipide enhanced the recovery of goblet cells after the vitrectomy was not determined in this study. Rios *et al.*^21^ reported that rebamipide induces the proliferation of cultured rat conjunctival goblet cells as determined by metabolic and histochemical methods. It has also been reported that rebamipide increases the expression of epidermal growth factor (EGF) and EGF receptors in regenerating glands of ulcer scars^27^, and EGFR signaling induces the expression of cyclooxygenase-2 (COX2) in gastric epithelial cells^28^,^29^. COX2-derived prostaglandin E2 stimulates mitogen-activated protein kinase which induces cell proliferation^30^,^31^. Thus, one possible explanation is that the postoperative topical rebamipide induced the proliferation of conjunctival goblet cells through the activation of the EGFR-COX2 signaling pathways.

Another possibility is that the increase of goblet cells after the vitrectomy might be related to the angiogenic action of rebamipide. It is known that the angiogenesis is essential for healing after tissue injury because regeneration of blood microvessels is a critical requirement for oxygen and nutrient delivery to the healing site. Tarnawski *et al.*^32^. showed that that rebamipide has two separate mechanisms with proangiogenic action; one through the activation of proangiogenic growth factor genes in gastric epithelial cells and also a direct angiogenic action on microvascular endothelial cells. A second possible mechanism is that the enhanced recovery of goblet cells after vitrectomy might be caused by accelerated wound healing due to the angiogenic action of rebamipide.

We studied not only patients with DM (Groups C and D), but also those without DM (Groups A and B) because we wanted to determine whether there is any difference in the effect of rebamipide in these types of patients. Our findings showed that the increase in the density of goblet cells after the vitrectomy was not statistically significant between the eye with and without rebamipide in DM patients (Fig. 4). We suggest that this might be due to an impairment of conjunctival blood flow in DM patients. Although rebamipide has cell-proliferation and/or angiogenic effects on damaged conjunctiva, these favorable effects might not be enough under the impaired conjunctival microcirculation in DM eyes.

There are four major limitations in this study. First, we did not measure the goblet cell density before the surgery because we wanted to minimize the trauma in these patients. However, without the baseline data of goblet cell density before the surgery, we could not determine the exact number of goblet cell that was reduced by the vitrectomy.

Second, we did not compare the symptoms between the four groups. Because topical rebamipide is known to improve the symptoms^23^,^24^,^25^, it might be useful to compare the scores of the symptoms between the eyes with and without topical rebamipide.

The third limitation is the short follow-up period. We measured the goblet cell density only at the end of surgery and 14 days after the surgery. However, the recovery of the goblet cell density might be more delayed especially in eyes with DM.

The fourth limitation is the small number of subjects (n = 10 for each group). The increment in the density of goblet cells might have attained significant values in eyes with rebamipide than without rebamipide in patients with DM if the number of subjects had been higher.

In conclusion, we showed that topical rebamipide can promote the recovery of conjunctival goblet cell density after vitrectomy. This suggests that the topical rebamipide can be helpful in treating patients with ocular surface damage during and after ocular surgery. Further clinical studies are needed for other ophthalmic surgeries including cataract or refractive surgeries.

## Additional Information

**How to cite this article**: Kato, K. *et al.* Effect of topical rebamipide on conjunctival goblet cell recovery after vitrectomy. *Sci. Rep.* **6**, 19516; doi: 10.1038/srep19516 (2016).

## Figures and Tables

**Table 1:** Demographic data of our four groups.

	DM (−)		*P*-value	DM		*P*-value
	Rebamipide (+)	Rebamipide (−)		Rebamipide (+)	Rebamipide (−)	
	Group A	Group B		Group C	Group D	
No. of eyes	10	10		10	10	
Age (yrs.)	65.8 ± 7.6	68.1 ± 9.8	0.57	63.4 ± 11.9	62.3 ± 4.7	0.79
Sex (Male/Female)	7/10	1/10	0.02*	2/10	0/10	<0.01*
HbA1c (%)				6.77 ± 0.88	7.55 ± 1.32	0.14
Duration of DM (yrs.)				17.0 ± 15.3	10.3 ± 8.25	0.24
Operation time (min.)	95.0 ± 32.3	87.3 ± 33.9	0.57	120.8 ± 49.9	110.7 ± 42.5	0.60
Combined PEA + IOL	9/10	10/10	1.00	6/10	7/10	1.00

Data are shown as the mean ± standard deviation. PEA + IOL, phacoemulsification and IOL implantation. *P < 0.05.

**Table 2:** Retinal diseases which required vitrectomy for each group.

	Group A	Group B	*P*-value	Group C	Group D	*P*-value
Epiretinal membrane	3	4		0	0	
Macular hole	6	4		0	0	
Retinal detachment	0	0		1	0	
Proliferative diabetic retinopathy	0	0		5	6	
Diabetic macular edema	0	0		4	4	
Others	1	2		0	0	
Total	10	10	0.65	10	10	0.58

Chi-square test was used between Group A and B, or Group C and D.
